# Recent advances of neoadjuvant chemoradiotherapy in rectal cancer: Future treatment perspectives

**DOI:** 10.1002/ags3.12213

**Published:** 2018-10-24

**Authors:** Kimihiro Yamashita, Takeru Matsuda, Hiroshi Hasegawa, Junko Mukohyama, Akira Arimoto, Tomoko Tanaka, Masashi Yamamoto, Yoshiko Matsuda, Shingo Kanaji, Tetsu Nakamura, Yasuo Sumi, Satoshi Suzuki, Yoshihiro Kakeji

**Affiliations:** ^1^ Division of Gastrointestinal Surgery Department of Surgery Kobe University Graduate School of Medicine Kobe Japan; ^2^ Division of Minimally Invasive Surgery Department of Surgery Kobe University Graduate School of Medicine Kobe Japan; ^3^ Division of International Clinical Cancer Research Department of Surgery Kobe University Graduate School of Medicine Kobe Japan

**Keywords:** adjuvant chemotherapy, chemoradiotherapy, lateral lymph node dissection, oxaliplatin, rectal cancer

## Abstract

Neoadjuvant chemoradiotherapy (nCRT) has been widely used as a multidisciplinary approach for stage II/III rectal cancer. However, its safety and efficacy are controversial because previous studies have shown conflicting outcomes. The present review aimed to elucidate the benefits and limitations of nCRT for patients with rectal cancer. Future perspectives of nCRT are also described. No recent randomized trials have been able to show a survival benefit, although many studies have demonstrated good local control with the use of fluoropyrimidine (e.g. 5‐fluorouracil [FU] or capecitabine)‐based nCRT. Addition of oxaliplatin (OX) to FU‐based nCRT might improve overall survival by preventing distant metastasis, as shown in recent meta‐analyses. However, control of adverse effects is an important concern with this treatment. New treatment strategies such as nonoperative management (watch and wait policy) and total neoadjuvant therapy (TNT) are promising, but the establishment of reliable diagnostic methods of metastasis is essential. Development of new biomarkers is also necessary to select patients who are more likely to benefit from nCRT.

## 1 INTRODUCTION

Postoperative local recurrence rate of rectal cancer is usually higher than that of colon cancer.^1^ In order to prevent local recurrence, a multidisciplinary approach is strongly required. In the narrow male pelvis, excess fat and the bulky neorectum must be excised completely for mid and low rectal cancers; consequently, technical difficulties arise in rectal cancer surgery. Moreover, anorectal, urinary, and sexual functions must be sufficiently preserved to maintain the patient's quality of life (QOL).^2^, ^3^ A multidisciplinary approach has been developed to secure curability and to preserve organ function. Neoadjuvant chemoradiotherapy (nCRT) plays a crucial role in rectal cancer treatment.

In many countries, CRT has long been a part of the standard treatment for rectal cancer. In Japan, preoperative treatment is not standard, and lateral lymph node dissection surgery was developed as a unique treatment.^4^ Recently, some hospitals have conducted clinical trials that assessed CRT. However, this standard treatment does not contribute significantly to overall survival (OS). Even if patients are fortunate enough to achieve long‐term survival, they suffer from complications as a result of the treatment. Recently, however, several studies on new methods related to CRT have been reported. The aim of this review was to understand the current status of nCRT in multidisciplinary treatments and to consider the future development of this treatment.

## 2 CURRENT STATUS OF NCRT

### 2.1 Evidence for its use as standard treatment

A multidisciplinary approach has been standardized for stage II/III rectal cancer in many countries.^5^ This approach consists of 5‐fluorouracil (5‐FU)‐based nCRT, radical surgery with total mesorectal excision (TME), and adjuvant chemotherapy (aCT).

Randomized clinical trials have confirmed that 5‐FU‐based nCRT improves local control compared to either neoadjuvant radiation therapy (nRT) alone or postoperative CRT.^6^, ^7^, ^8^ However, adding 5‐FU to radiation therapy (RT) does not improve disease‐free survival (DFS) and OS over 10 years of follow up.^9^ Conversely, in NSABP‐R03, capecitabine and RT significantly improved DFS and showed a trend toward improved OS compared with postoperative CRT. However, we must note that this trial was not restricted to TME surgery.

No recent randomized trials on rectal cancer treatments that used either short‐course nRT alone or nRT combined with 5‐FU have shown a survival benefit, even after a follow up of more than 10 years for the German CAO/ARO/AIO‐94 trial and the Dutch TME trial.^10^, ^11^ In conclusion, 5‐FU‐based nCRT does not contribute positively to long‐term outcomes and OS.

### 2.2 Role of nCRT in the multidisciplinary approach

#### 2.2.1 Adaptation of nCRT

Accurate imaging of the tumor and lymph nodes is essential to determine the staging of rectal cancer. In addition to clinical examination, endoscopy, and screening for distant metastases, the treatment strategy is based on pretherapeutic imaging and is currently guided by assessment of the risk of local recurrence based on European Society for Medical Oncology (ESMO) and National Comprehensive Cancer Network (NCCN) guidelines.^5^, ^12^ In the ESMO guidelines, pretherapeutic local imaging with magnetic resonance imaging (MRI) is indispensable for staging. In terms of T category, using high‐quality MRI helps accurately evaluate the tumor extent. For example, based on the mesorectal depth of invasion beyond the muscularis propria, cT3 rectal cancer can be further classified into subgroups (T3a, <1 mm; T3b, 1‐5 mm; T3c, 5‐15 mm; and T3d, >15 mm). Herein, the recommended treatment options are strictly determined according to the risk category. MRI also allows precise assessment of the status of mesorectal fascia (MRF) such as distance from a tumor to the MRF (circumferential margin: CRM) and the presence of extramural vascular invasion (EMVI). For a comprehensive evaluation of the risk category in rectal cancer without metastasis, besides T and N stages, EMVI, MRF involvement, patient characteristics, and patient preferences are considered. In rectal cancers classified as intermediate risk, which is “cT3a/b very low, levators clear, MRF clear, cT3a/b in mid or high rectum, cN1‐2 (not extranodal), no EVMI”, giving a short‐course of nRT or nCRT can maintain the TME plane in surgery. In surgical procedures, the TME plane must be maintained. The majority of local recurrences historically reflect inadequate TME.^13^ Chen et al showed a lower risk of a positive CRM and higher quality TME in patients undergoing nCRT.^14^ In conclusion, nCRT can guarantee the quality of surgery.

#### 2.2.2 Optimal interval from the end of nCRT to surgery

Currently, the interval from the end of nCRT to surgery is based on the Lyon R 90‐01 trial.^15^ This trial demonstrated that nRT increased the rate of a pathological complete response (pCR) or near pCR from 10.3% at 2‐week intervals to 26% at 6‐ to 8‐week intervals (*P *=* *0.0054). Thus far, the optimal interval is considered to be 6‐8 weeks. The rationale for this interval is that it is expected to increase the pCR rate and to reduce postoperative complications. In a systematic review, Du et al analyzed 13 studies involving 19 652 patients to elucidate how the interval between nCRT and surgery affects the pCR rate.^16^ They showed that compared to an interval of ≤8 weeks, carrying out surgery after an interval of ≥8 weeks after the end of nCRT is safe and efficacious for patients with rectal cancer, and it significantly improves the pCR rate without increasing operative time or the incidence of postoperative complications.

Recently, an open‐label randomized controlled phase III trial (GRECCAR ‐ 6) examined the effect of the interval between the end of nCRT and surgery (7 vs 11 weeks) on the pCR rate. Surprisingly, no difference in the pCR rate was observed between the groups (15.0% vs 17.4%; *P *=* *0.5983 in the intent‐to‐treat analysis and 5.7% vs 17.2%; *P *=* *0.7800 in the per‐protocol analysis). All postoperative complications were significantly higher in the 11‐week group than in the 7‐week group (32% in the 7‐week group vs 44.5% in the 11‐week group; *P *=* *0.04). These included medical complications and delayed perineal wound healing after abdominoperineal resection (APR).^17^ This work provides new evidence that a longer interval between nCRT completion and surgery did not improve the pCR rate.

### 2.3 Attempt to improve the regimen

Based on the efficacy shown in colon cancer trials, oxaliplatin (OX), in combination with fluoropyrimidine (e.g. 5‐FU or capecitabine)‐based regimens and RT, is expected to both enhance primary tumor shrinkage and reduce micro metastases at distant sites for rectal cancer. The regimen including OX in nCRT has been tested in several large phase III studies (Table 1).^18^, ^19^, ^20^, ^21^, ^22^, ^23^, ^24^, ^25^, ^26^, ^27^, ^28^, ^29^

**Table 1:** Phase III trial adding oxaliplatin to neoadjuvant chemoradiotherapy for rectal cancer

Study name	Country year	Study arm	No. of patients	Primary endpoint	3y OS	3y DFS	Local recurrence (%)	Distance metastasis (%)	pCR rate (%)	Grade 3/4 toxicities (%)	Sphincter‐saving surgery (%)	Compliance with preoperative chemoradiotherapy in oxaliplatin groups (%)
CAO/ARO/AIO‐04^18^, ^19^	Germany 2012, 2015	5‐FU	623	DFS	88.0	71.2	4.6	6.0	13.0	13.0	88.0	–
		5‐FU + OX^a^	613		88.7 (HR 0.96, CI 0.72‐1.26)	75.3 (HR 0.79, CI 0.64‐0.98)	2.9	4.0 (all sites)	17.0 (*P* = 0.031)	17.0 (*P* = 0.04)	88.0	85.0
ACCORD 12^20^, ^21^	France 2012	Cape	299	pCR	87.6	67.9	6.1	4.2 (abdominal)	13.9 (P < 0.001)	10.9 (*P* < 0.001)	–	–
		Cape + OX^a^	299		88.3 (HR 0.94, CI 0.59‐1.48)	72.7 (HR 0.88, CI 0.65‐1.18)	4.4	2.8	19.2	25.4	–	41.0
STAR‐01^22^	Italy 2011	5‐FU	379	OS, pCR as protocol‐planned comparative analysis	–	–	6.0	2.9 (abdominal)	16.4	8.0 (*P*< 0.001)	80.6	–
		5‐FU + OX	368		–	–	1.3	0.5	16.8 (P = 0.904)	24.0	81.7	66.0
PETACC‐6^23^, ^24^	Germany 2014, 2018	Cape	623	DFS	83.1^b^	71.3^b^	–	–	11.3	15.2	70.0	–
		Cape + OX	613		80.1^b^ (HR 1.17, *P* = 0.25)	70.5^b^ (HR 1.02, *P* = 0.84)	–	–	13.3	36.7	65.1 (*P* = 0.09)	–
NSABP R‐04^25^, ^26^	USA 2014, 2015	5‐FU/Cape	949	Locoregional failure, sphincter‐saving surgery	79.0^b^	64.2^b^	12.1	–	17.8	6.6	62.0	–
		5‐FU/Cape + OX^a^	659		81.3^b^ (HR 0.89, *P* = 0.38)	69.2^b^ (HR 0.91, *P* = 0.34)	11.2 (*P* = 0.7)	–	19.5 (*P* = 0.42)	15.4 (*P* < 0.0001)^c^	62 (*P* = 0.28)	–
JIAO 2015^27^	China 2015	Cape	103	DFS, OS	86.4	70	5.8	28.2	23.3	6.8	77.7	–
		Cape + OX^a^	103		90.3 (*P* = 0.515)	80.6 (*P* = 0.076)	4.9 (*P* = 0.7)	16.5 (*P* = 0.045)	19.4 (*P* = 0.479)	16.5 (*P* = 0.03)	84.5	81.6
FOWARC^30^, ^31^	China 2016, 2018	5‐FU	155→130	DFS	76.4 ± 3.8	93.7 ± 2.2	10.0	–	14.0	10.7	84.4	–
		5‐FU + OX	158→142		77.8 ± 3.5	92.0 ± 2.3	8.5	–	27.5 (*P* = 0.05)	21.4 (*P* = 0.037)	87.2	94.9

5‐FU, 5‐fluorouracil; ACCORD 12, Actions Concertées dans les Cancers Colorectaux et Digestifs; CAO/ARO/AIO‐04, Chirurgische Arbeitsgemeinschaft für Onkologie/Arbeitsgemeinschaft Radiologische Onkologie/Arbeitsgemeinschaft Internistische Onkologie; Cape, capecitabine; CI, 95% confidence interval; DFS, disease‐free survival; FOWARC, The Neoadjuvant FOLFOX6 Chemotherapy With or Without Radiation in Rectal Cancer study; HR, hazard rate; NSABP R‐04, National Surgical Adjuvant Breast and Bowel Project trial R‐04; OS, overall survival; OX, oxaliplatin; pCR, pathological complete regression; PETACC‐6, the Pan‐European Trials in Alimentary Tract Cancer; STAR‐01, Studio Terapia Adiuvante Retto..

^a^OX‐weekly regimen, ^b^5 y, ^c^grade 3‐4 diarrhea only.

There are five major randomized trials that determine whether the addition of OX to 5‐FU/capecitabine‐based nCRT offers an advantage compared with 5‐FU‐based nCRT. In contrast to the German CAO/ARO/AIO‐04 trial, the results from the ACCORD 12, STAR‐01, PETACC‐6, and NSAPB R‐04 trials failed to show a significant improvement in the primary endpoints with the addition of OX.^20^, ^21^, ^22^, ^23^, ^24^, ^25^, ^26^ Some researchers believed that the DFS benefit in the German CAO/ARO/AIO‐04 trial was due to the adaptation of OX‐based aCT regimens.^18^, ^19^, ^30^, ^31^ This regimen was different from mFOLXOX6; the dose regimen for every 2 weeks (days 1 and 15) was as follows: OX 2‐hour infusion of 100 mg/m^2^, Leucovorin (Folinic Acid) 2‐hour infusion of 400 mg/m^2^, and 5‐FU 46‐hour infusion of 2400 mg/m^2^ starting. In addition, NSABP‐R04 showed that capecitabine could replace 5‐FU in aCT or nCRT regimens.^25^, ^26^ Considering these results from five trials in European countries and the USA, most oncologists recommend continuous infusion of 5‐FU or oral capecitabine without OX for nCRT.

Furthermore, two major trials were carried out in China to evaluate whether the addition of OX to FU‐based nCRT has an advantage compared with FU‐based nCRT. The JIAO2015 trial did not significantly improve OS and DFS but reduced the rate of distant metastasis.^27^ The FOWARC trial was a multicenter open‐label randomized phase III study. The patients were divided into three groups: 5‐FU‐RT, mFOLFOX 6‐RT, and mFOLFOX 6 groups. The trial aimed to examine the additive effect of OX in nCRT and safety and efficacy without RT in neoadjuvant chemotherapy in the mFOLFOX 6 group. Compared with the 5‐FU‐RT group, the mFOLFOX 6‐RT group had a higher pCR rate (27.5% vs 14.0%, OR = 0.428, 95% CI: 0.237‐0.776, *P *=* *0.005) and higher but acceptable toxicities.^28^, ^29^

These seven, representative trials must be verified in detail. Yang et al carried out a meta‐analysis considering a recent update of the aforementioned results,^32^ which showed that the FU‐based nCRT with OX group showed a marginally significantly higher DFS than the FU‐based nCRT group and had a significantly decreased distant metastasis rate. Furthermore, the FU‐based nCRT with OX group showed a significantly increased pCR rate compared with the FU‐based nCRT group. In addition, regarding the dose intensity of OX in CRT, the trials CAO/ARO/AIO‐04, JIAO2015, and FOWARC showed higher compliance rates, which showed some favorable outcomes. The relatively good tolerance observed in these trials prompted us to conclude that the addition of OX can be a new treatment option. Moreover, it is important to know how to properly use OX. A weekly OX regimen is used as a radio‐sensitizing agent and not as a normal chemotherapeutic agent. In five large phase III studies shown in Table 1, the additive OX is used as a weekly regimen. De Felice et al showed that adding weekly OX to FU in nCRT appeared to moderately increase the pCR rate and reduced the rate of intra‐abdominal or perioperative metastases.^33^ However, the precise role of OX in nCRT remains unclear.

Additionally, apart from being combined with OX, FU‐based nCRT regimens have been combined with irinotecan and molecular targeted agents, such as vascular endothelial growth factor (VEGF) inhibitors, epidermal growth factor receptor (EGFR) inhibitors, and multi‐kinase inhibitors, in phase I‐III trials of nCRT. Early phase I‐II trials with these agents suggested higher pCR rates compared with FU‐based nCRT.^34^, ^35^ Recently, researchers have attempted to develop a treatment that considers the state of *KRAS/RAS* mutations.^36^, ^37^ However, for these agents, an increased pCR rate was associated with increased acute toxicity.^38^ Thus, further investigations are warranted for developing new nCRT regimens.

## 3 SURGICAL MANAGEMENT AFTER NCRT

### 3.1 Anus‐preserving surgery formula

Many studies have shown that nCRT does not increase the frequency of postoperative complications or, in cases of complication occurrence, they were well tolerated.^19^, ^39^, ^40^ Intersphincteric resection (ISR) is an anus‐preserving surgery and an alternative to APR for low‐lying rectal cancer within 5 cm from the anal verge. Theoretically, ISR procedures after nCRT were expected to increase the rate of anus preservation. Lee et al showed that ISR following nCRT could be feasible in patients with pathological (p)‐stage I/II low‐lying rectal cancer, but, based on a retrospective analysis, it might be related to poor oncological outcomes in those with p‐stage III disease.^41^ They reported that 3‐year DFS (p‐stage 0, 96.2%; I, 84.8%; II, 72.9%; III, 38.0%) and 3‐year local recurrence‐free survival (LRFS) (p‐stage 0, 100.0%; I, 92.4%; II, 91.1%; III, 70.9%) depended on p‐tumor‐node‐metastasis (TNM) stages, which showed poor prognosis in p‐stage III. Based on these data, they concluded that the indication for ISR or APR should be carefully evaluated in cases of low‐lying rectal cancer with preoperative staging after nCRT. Saito et al suggested caution while carrying out ISR after nCRT because a group that underwent ISR after CRT showed significantly worse quality of life regarding anorectal function and mental state.^42^ In a recent systematic review, oncological outcomes after ISR for low‐lying rectal cancer are acceptable, but they had imperfect anorectal functional results showing that 29.1% (95% CI: 15.3‐43.0) of all patients experienced fecal soiling and the rate of incontinence to flatus was 23.8% (95% CI: 6.7‐30.5).^43^

### 3.2 Lateral lymph node dissection after nCRT

Recent studies suggest that lateral pelvic lymph node (LLN) metastasis is a major cause of local recurrence in patients with lower rectal cancer, even when treated with nCRT.^44^, ^45^ In Japan, mesorectal excision (ME) and lateral pelvic lymph node dissection (LLND) surgery without nRT/CRT has been the standard procedure.^4^ In Western countries, the presence of lateral lymph node metastasis is considered a systemic disease. Therefore, LLND has been criticized, and it is not widely accepted. Georgiou et al suggested that LLND did not confer a significant survival benefit, but it did seem to be associated with increased urinary and sexual dysfunction.^46^ Recently, The Japan Clinical Oncology Group (JCOG) conducted the JCOG0212 trial, which aimed to confirm the noninferiority of ME alone (experimental arm) to ME with LLND (standard arm) in terms of efficacy. The results indicated that the local recurrence rate was significantly higher in the ME‐alone group than in the ME with LLND group (12.6% vs 7.4%, *P *=* *0.024), although there was no difference in 5‐year relapse‐free survival (73.3% vs 73.4%, *P *=* *0.0547).^47^ As a result, ME and LLND surgery remain the standard procedure. In addition, this study interestingly indicated that urinary dysfunction was not directly associated with the LLND procedure but was directly associated with tumor location and blood loss.^48^

Based on these results, some investigators carried out selective LLND after nCRT where LLND was carried out only for patients with clinically positive LLN based on pretreatment images.^49^, ^50^, ^51^ Matsuda et al reported no local recurrence in the LLND (−) group regardless of the histological response, suggesting that nCRT sufficiently suppresses local recurrence in patients with clinically negative LLN. Importantly, in their study, the 5‐year LRFS was only 66.9% in pathological poor responders to nCRT whereas it was 100% in good responders.^49^ Akiyoshi et al demonstrated that the recurrence rate at LLN after nCRT was 3.4% and 0% in the LLND (−) and LLND (+) groups, respectively, and that LLN might improve the local control and survival of patients with LLN metastasis in low rectal cancer treated with nCRT.^50^ Oh et al suggested that the decision to carry out LLND should be based on the lateral lymph node response to nCRT. Persistent LLN >5 mm observed using post‐nCRT MRI were significantly associated with residual tumor metastasis, unlike responsive LLN after nCRT (short‐axis diameter ≤5 mm) [pathologically, 61.1% (22 of 36) vs 0% (0 of 30), *P *<* *0.001].^52^ The indications for LLND following nCRT, which would consequently improve local and systemic control, should be further discussed.

### 3.3 Watch and wait policy

In a pooled analysis, approximately 16% of patients who underwent nCRT for locally advanced rectal cancer showed a pCR after a standard resection.^53^ Habr‐Gama et al proposed the watch and wait policy, in which patients with a clinical complete response (cCR) were followed up with no operation and close surveillance including physical examination, endoscopy, and imaging.^54^ This approach has been widely used as an acceptable strategy in some guidelines.^12^, ^55^ Habr‐Gama et al reported the long‐term outcomes of 71 patients with rectal cancer with a cCR who were followed by a watch and wait policy (mean follow up, 57 months).^54^ On initial assessment, 26.8% of the patients who underwent CRT had cCR. The 5‐year OS and DFS rates were 100% and 92% compared with 88% and 83% in incomplete responders treated with TME salvage surgery, respectively. In another report, a propensity‐score matched cohort study from the UK followed 129 patients with cCR compared with patients without cCR who underwent surgical resection.^56^ Of the 129 patients monitored using the watch and wait policy (median follow up, 33 months), 44 (34%) patients showed local regrowth (3‐year actuarial rate, 38%); 36 (88%) of 41 patients with non‐metastatic local regrowth were salvaged. In the matched analyses (109 patients in each treatment group), no differences were noted in the 3‐year non‐regrowth DFS (88% with watch and wait vs 78% with surgical resection) and 3‐year OS (96% vs 87%, respectively) rates.

Currently, there are no known prediction factors that determine which patients will respond to CRT based on pretherapeutic variables such as gender, age, N stage, and tumor location.^57^ Lymph node pathological status after CRT (ypN) was significantly associated with the risk of local recurrence and subsequent distant metastases.^58^ Reliable methods are crucial to identify patients with cCR and to follow up when implementing the watch and wait policy. Digital rectal examination and endoscopy are generally used to assess cCR and local recurrence.^59^, ^60^, ^61^ Lambregts et al reported that adding diffusion‐weighted imaging (DWI) improved the sensitivity of MRI for diagnosing local tumor regrowth.^62^ As previously described, lymph node status was the most important prognostic factor after CRT in rectal cancer. Although MRI is widely used and is recommended to assess lymph node involvement, a systematic review reported that lymph node assessment was poor using MRI.^63^, ^64^ Moreover, in a pooled analysis, even DWI‐MRI or positron emission tomography (PET) with ^18^F‐labeled fluoro‐2‐deoxyglucose (^18^F‐FDG) and computed tomography (CT) (^18^F‐FDG‐PET/CT) had a low positive‐predictive value for predicting pCR and was not accurate enough to safely select patients for organ‐sparing strategies.^65^ Currently, based on a systematic review, functional MRI such as dynamic contrast‐enhanced (DCE)‐MRI following PET/CT showed high diagnostic accuracy and the results were also more reliable than conventional MRI and DWI alone.^66^ The uncertainty of these diagnostic methods has been a barrier to the watch and wait approach. There are current studies ongoing to evaluate whether these examinations are acceptable as standard methods.

## 4 ADJUVANT CHEMOTHERAPY AFTER NCRT FOLLOWED BY SURGERY

Adjuvant chemotherapy in colon cancer reduces the incidence of distant relapse and improves OS. With this same argument, aCT was incorporated into multimodal treatment strategies in rectal cancer. However, although aCT after nCRT followed by surgery is currently recommended in NCCN guidelines, in a randomized trial, the beneficial contribution of aCT in these strategies has not yet been clearly shown.^5^ It remains difficult to clarify the actual role of aCT for rectal cancer after nCRT.^9^ Several studies have attempted to address the role of aCT in resected rectal cancer following nRT or nCRT. EORTC 2291 was a large randomized trial with this aim. The trial used a 2 × 2 factorial design that randomized 1011 patients to nRT or nCRT and, in a second randomization, to aCT or observation. The aCT regimen was 5‐FU‐based (5‐FU and leucovorin). No significant difference in 10‐year OS was observed between the patients who received nCRT with or without aCT (51.8% vs 48.4%, *P *=* *0.32).^9^ In addition, a systematic review showed that an FU‐based aCT after nCRT did not improve DFS or OS and distant metastasis.^67^ However, in the ADORE trial and the German CAO/ARO/AIO‐04 study, 5‐FU or capecitabine with OX as a regimen for locally advanced rectal cancer showed a superior DFS compared to 5‐FU or capecitabine alone.^19^, ^68^

Conversely, in rectal cancer treatment, there are two major problems to consider while investigating the role of aCT. The first is the poor compliance of aCT. Although compliance with aCT in colon trials has been reported to be 70%‐86%, in most rectal trials, aCT compliance has reduced to 43%‐58%.^69^ The second is the responsibility to nCRT. After nCRT, the pCR rate observed is approximately 15%, with a further 20% of patients being downstaged to ypT1/T2N0. However, there is no clear consensus regarding whether patients with pCR should undergo aCT because the prognosis of these treatments is favorable, but data supporting their use are limited.^70^ Polanco et al demonstrated that aCT was associated with improved OS in patients with pCR after nCRT, as reported in the National Cancer Database between 2006 and 2012 by propensity‐score matching.^71^ However, patients who achieve a pCR or a clinically significant downstage to ypT1/T2N0 after chemoradiation usually have an excellent prognosis and are unlikely to benefit from further CT.^72^

## 5 FUTURE OF NCRT

### 5.1 Concept of total neoadjuvant therapy

Distant metastatic disease remains the most significant cause of death in patients with locally advanced rectal cancer even though the rates of local recurrence have been markedly decreased by improved surgical techniques and CRT implementation. The concept of total neoadjuvant therapy (TNT), in which CRT and CT are given prior to surgery, has been developed as an effective systemic therapy to improve long‐term survival.^73^ As described above, trials evaluating aCT for rectal cancer had disadvantages such as poor compliance rates and incompatible survival results.^69^ Shifting systemic therapy to the neoadjuvant setting has the promise to improve compliance rates, reduce toxicities, and decrease distant relapse rates. Cercek et al demonstrated that patients in the TNT cohort received greater percentages of the planned OX and FU doses than those in the control cohort and that the CR rate, including both pCR and cCR for at least 12 months, was 36% in the TNT cohort compared with 21% in the control cohort. This study suggested that TNT facilitated the delivery of planned systemic therapy.^74^ NCCN guidelines categorize TNT as a viable treatment strategy for rectal cancer. Ongoing phase II and III trials are now assessing the long‐term disease‐related outcomes of TNT. In addition to improving survival, TNT has the potential to increase the population of patients with rectal cancer who are eligible for organ preservation.

### 5.2 Biomarker for precision medicine

There is a critical need to identify biomarkers to help select patients who are more likely to benefit from CRT and to prevent patients from receiving the toxicity associated with ineffective CRT.

#### 5.2.1 Mutations in KRAS and TP53

Mutations in *KRAS* and *TP53* that are frequently observed in colorectal cancer have been considered to be the markers of a poor response to CRT in rectal cancer.^75^, ^76^ Duldulao et al carried out *KRAS* and *TP53* genotyping in rectal cancer and showed that tumors with the *KRAS* mutation were less likely to achieve pCR than those with wild‐type *KRAS* (*P *=* *0.006).^77^ Interestingly, in their study, no tumors with *KRAS* codon 13 mutations achieved pCR (*P *=* *0.03), and these tumors also had a higher incidence of the *TP53* mutation compared with tumors with other *KRAS* mutations (*P *=* *0.02). These findings suggest that mutations in different *KRAS* codons may have different effects on the resistance of rectal cancer to CRT and that the rectal cancers carrying *KRAS* and *TP53* mutations are less likely to respond to CRT compared with wild‐type tumors. Reportedly, expression levels of several tissue‐based proteins, including EGFR, VEGF, Ki‐67, p21, p53, Bcl2, COX‐2, hypoxia‐inducible factor 1‐alpha (HIF1‐α), matrix metalloproteinase (MMP)‐2, MMP‐9, DUOX2, AKT, DEK, Pim‐3, and FN1, are associated with response to nCRT; however, further prospective studies are necessary to validate the utility of these predictive biomarkers.^76^, ^78^, ^79^, ^80^, ^81^, ^82^, ^83^

#### 5.2.2 Serum carcinoembryonic antigen levels

More than half of the reported studies used 5 ng/mL as the cutoff serum carcinoembryonic antigen (CEA) value. Pre‐nCRT CEA levels were independently associated with a poor pCR rate, reduced pathological tumor regression, reduced tumor downstaging, and decreased OS in several patients with locally advanced rectal cancer.^76^, ^84^ Other studies have also shown a correlation between post‐CRT CEA levels and pCR.^85^ These retrospective studies suggest that low pre‐CRT and post‐CRT CEA levels might be useful in predicting pCR and better patient outcomes.

#### 5.2.3 Systemic inflammatory response

A high modified Glasgow prognostic score (mGPS), high neutrophil‐to‐lymphocyte ratio, low platelet‐to‐lymphocyte ratio, and low lymphocyte‐to‐monocyte ratio have been associated with a poor response to CRT.^86^, ^87^

#### 5.2.4 microRNAs

microRNAs (miRNAs) can be promising predictive markers. miRNAs are non‐coding RNAs with <25 nucleotides that regulate various cell functions in colorectal cancer.^88^ Yu et al carried out a global miRNA analysis in CRT‐sensitive and ‐resistant patients and found that miR‐345 was significantly elevated in CRT‐resistant patients (*P *=* *0.002).^89^ Some other miRNAs, including miR‐194, ‐145, ‐21, ‐125b, and ‐143, have been reported to be the predictive biomarkers of CRT.^90^, ^91^, ^92^, ^93^ None of them has been clinically assessed; however, it is possible that the optimal combination of these miRNAs can act as reliable biomarkers of a response to CRT.

#### 5.2.5 Circulating tumor DNA

Tumor‐specific DNA can be detected in the peripheral blood (circulating tumor DNA: ctDNA) of patients with colorectal cancer or other solid tumors. Monitoring ctDNA enables continuous collection of genetic information with mild invasion, possibly overcoming intratumoral temporal and spatial heterogeneity. In the EXPERT‐C trial, digital droplet PCR (ddPCR) was used to investigate mutations in specific genes such as *KRAS/BRAF* in ctDNA from baseline blood samples of patients with rectal cancer who were treated with capecitabine with OX followed by nCRT, surgery and capecitabine with OX ± cetuximab as aCT.^94^ In conclusion, the detection of a *KRAS* mutation in ctDNA failed to predict prognosis or refine patient selection for cetuximab. Conversely, a successful example is reported to examine the presence of ctDNA as a prognostic factor.^95^ This study was designed to collect plasma samples from patients with locally advanced rectal cancer planned for nCRT at the status of pre‐CRT, post‐CRT, and 4‐10 weeks after surgery. Significantly, worse recurrence‐free survival was observed if ctDNA was detectable after nCRT (*P *<* *0.001) or after surgery (*P *<* *0.001). Postoperative ctDNA detection predicted recurrence irrespective of the use of adjuvant chemotherapy. Postoperative ctDNA status remained an independent predictor of recurrence‐free survival. Future developments in ctDNA analysis are highly expected in rectal cancer.

### 5.3 Compatibility of immunotherapy with nCRT

The emergence of checkpoint inhibitor based‐immunotherapy has rendered it necessary to evaluate the effects of combinations with other therapies.^96^ Among them, combination therapy of nCRT with a checkpoint inhibitor is expected to be promising. Previous studies have suggested that the effect of radiotherapy at a locoregional tumor site can lead to regression of metastatic cancer at distant sites out of the irradiated field (the abscopal effect); this phenomenon has been correlated with mechanisms involving the immune system.^97^ Moreover, in the preclinical model, immunotherapy tends to be more effective in preoperative settings than in postoperative settings.^98^ An analysis of surgical specimens demonstrated that nCRT affects the intratumor immunological microenvironment.^99^, ^100^ Kalanxhi et al reported that FU‐based TNT with OX generates an immune response and contributes to survival without distant metastasis in a phase II single‐arm study. The immune response was assessed by studying the circulating levels of the fms‐like tyrosine kinase 3 ligand (Flt3L) protein, which is a factor reflecting both therapy‐induced myelosuppression and tumor antigen‐presenting dendritic cells activation.^101^ Based on these considerations, several phase II clinical trials combining immune checkpoint inhibitors with CRT have been initiated for rectal cancer (NCT02586610 and NCT02948348). Results of these trials will help implement strategies improving long‐term survival.

## 6 CONCLUSION

Chemoradiotherapy has been a part of the standard multidisciplinary approach for rectal cancer treatment. The effect of OX has been clarified in the treatment. In nCRT, the addition of OX as a radio‐sensitizing agent to FU‐based nCRT may contribute to preventing distant metastases. Moreover, in aCT after nCRT, an FU‐based regimen with OX is clearly effective. Furthermore, the next‐generation therapies based on CRT, such as LLND surgery, watch and wait policy, and TNT are being assessed in a clinical trial with high expectations. We hope that these results can provide benefits for patients with rectal cancer.

## DISCLOSURE

Conflicts of interest: Authors declare no conflicts of interest for this article.
